# Combination of albumin-to-globulin ratio and plasma fibrinogen is a sensitive tool for preoperative screening of infected nonunion in patients undergoing reoperation after open reduction and internal fixation: a retrospective study

**DOI:** 10.1186/s13018-022-03363-3

**Published:** 2022-10-29

**Authors:** Zhen Wang, Haijun Mao, Guangyue Xu

**Affiliations:** Department of Orthopedic Surgery, Nanjing Drum Tower Hospital, The Affiliated Hospital of Nanjing University Medical School, Nanjing, China

**Keywords:** Plasma fibrinogen, Albumin-to-globulin ratio, Preoperative diagnosis, Infected nonunion

## Abstract

**Background:**

Accurate preoperative diagnosis of infected nonunion remains a challenge. Here, we evaluated the diagnostic potential of novel biomarkers for infected nonunion.

**Methods:**

A cohort of 275 patients who underwent surgery for suspected septic nonunion after open reduction and internal fixation were enrolled. Preoperatively analyzed clinical parameters included white blood cell (WBC) count, C-reactive protein (CRP), erythrocyte sedimentation rate (ESR), albumin, globulin, albumin-to-globulin ratio (AGR), plasma D-dimer, plasma fibrinogen, platelet count (PC), monocyte-lymphocyte ratio (MLR), neutrophil–lymphocyte ratio (NLR), and platelet-to-lymphocyte ratio (PLR). Receiver operating characteristic (ROC) curves, sensitivity, and specificity were utilized to compare the diagnostic potential of those biomarkers.

**Results:**

The WBC count and levels of CRP, ESR, NLR, MLR, PLR, PC, plasma D-dimer, plasma fibrinogen, and globulin in infected nonunion patients were significantly higher (*p* < 0.05) than those in aseptic patients. The albumin and AGR levels of the infected nonunion group were significantly lower (*p* < 0.05) than the aseptic group. The ROC curve analysis showed that the diagnostic accuracy of AGR and plasma fibrinogen was good. The combination of AGR with plasma fibrinogen had the highest area under the curve (AUC) (0.916). The sensitivity and specificity were 70.27% and 91.04% for AGR, and 67.57% and 84.08% for plasma fibrinogen, respectively. The combination of AGR with plasma fibrinogen showed a sensitivity of 86.49% and specificity of 92.54%. In patients with comorbidities, the diagnostic accuracy of the combination of AGR with plasma fibrinogen was also good.

**Conclusions:**

AGR and plasma fibrinogen are promising biomarkers to improve the diagnosis of infected nonunion. The combination of AGR with plasma fibrinogen is a sensitive tool for screening infected nonunion.

## Background

Infected nonunion is one of the most challenging complications after open reduction and internal fixation (ORIF) for orthopedists. Ineffective control of infected nonunion can potentially result in higher hospitalization costs, longer treatment course, and higher morbidity and mortality rates than the primary procedure [1–3]. According to the 2018 Fracture-related Infection Consensus Definition [3], the gold standards for diagnosis of infected nonunion are histology and microbiology of deep tissue specimens, which are only available postoperatively and hence have no preoperative diagnostic value. Tibial fractures are the most frequent sites of nonunion and infected nonunion among limb fractures after ORIF [4–6]. Moreover, it is usually difficult to accurately differentiate between infected nonunion and aseptic nonunion preoperatively, especially when confirmatory clinical features such as a sinus tract and visible purulent discharge are absent [7, 8]. Thus, developing a non-invasive method that is more convenient, tolerable, reliable, economical, and faster for preoperatively diagnosing infected nonunion is of great importance, which can substantially influence treatment plans [9, 10].

Currently, white blood cell (WBC) count, erythrocyte sedimentation rate (ESR), and C-reactive protein (CRP) are cheap, easy to use, and widely available traditional blood inflammatory markers, which can provide preoperative information for diagnosing infection [11, 12]. These biomarkers are usually elevated in acute infected nonunion, but mostly remain normal in late or chronic infections, or are influenced by other infections or inflammation [13]. These blood markers showed poor sensitivity and specificity for the preoperative diagnosis of infected nonunion in prior studies [14, 15]. Therefore, new biomarkers for preoperatively diagnosing infected nonunion are particularly important, which can substantially affect treatment plans [16].

Coagulation-related parameters such as fibrinogen, D-dimer, and platelet count (PC) have been reported to be promising diagnostic markers of infection, and some studies have proved that fibrinogen, D-dimer, or PC are useful in the diagnosis of periprosthetic joint infection (PJI) [17–19]. Moreover, serum D-dimer and plasma fibrinogen have shown good performance in the diagnosis of infected nonunion in past studies [20, 21]. Alternative monocyte-lymphocyte ratio (MLR), neutrophil–lymphocyte ratio (NLR), and platelet-to-lymphocyte ratio (PLR) have been strongly associated with inflammation in many diseases such as hepatitis virus infection, rheumatic diseases, and infective endocarditis [22, 23]. Albumin is the main protein in human serum, and hypoalbuminemia, a historic index of malnutrition, has recently been associated with infection in orthopedics [24–26]. Globulin, which is a component of complements and ceruloplasmin, generally increases during the inflammatory process [27]. Hence, the albumin-to-globulin ratio (AGR), which considers both albumin and globulin levels, is negatively associated with chronic inflammation [28]. Furthermore, several studies have reported that globulin and AGR were useful biomarkers in the diagnosis of PJI [29, 30]. However, a comparison of the accuracy of all these blood biomarkers in diagnosing infected nonunion after internal fixation is still not available.

Therefore, this retrospective study was performed to assess the ability of WBC, CRP, ESR, albumin, globulin, AGR, plasma D-dimer, plasma fibrinogen, PC, MLR, NLR, and PLR to diagnose infected nonunion in patients undergoing reoperation after internal fixation and to compare the diagnostic performance of these ratios with those of WBC, CRP, and ESR.

## Materials and methods

We conducted a retrospective study in which data on patients admitted to our hospital between January 2017 and February 2022 were analyzed. Ethical approval was obtained from the Clinical Research Ethics Committee of The Affiliated Drum Tower Hospital of Nanjing University Medical School. Our hospital’s institutional review board approved the study (2022–023) and waived the requirement for written informed consent because it was retrospective, involved only anonymized patient data, and posed no risk to patients [31]. The inclusion criteria were as follows: (1) patients aged ≥ 18 years and (2) those with nonunion after ORIF that required reoperation. The exclusion criteria were as follows: (1) age < 18 years (*n* = 3), (2) without complete blood workup (*n* = 7), and (3) unavailability of complete examinations (*n* = 4). Patients with nonunion after a joint fusion (*n* = 10), corrective osteotomy (*n* = 6), and pathological fracture (*n* = 3) were also excluded. Patients on antibiotic therapy 2 weeks prior to the reoperation (*n* = 11), and those with related comorbidities (*n* = 36) were also excluded. Finally, a total of 275 patients who underwent reoperation for a suspected infected nonunion were eligible for inclusion.

The clinical features of patients with nonunions were comprehensively interpreted by the attending physician after admission. Baseline demographic features, including age, sex, BMI, smoking, and fracture position were collected. A nonunion was determined as radiographic evidence of nonprogression of healing for at least 3 months, or lack of healing by 9 months since the initial injury [32]. According to the 2018 Infected Nonunion Consensus Definition [3], information on demographics, histological, and microbiological results from intraoperative sampling; visible pus; sinus tract; and serum inflammatory markers were recorded. The presence of an active infection was either confirmed by microbial detection in tissue and joint samples, or in the case of negative microbial detection, by the presence of a soft tissue defect with an exposed plate and existing joint sinus. The 275 patients were divided into two groups: 74 patients with infected nonunion and 201 aseptic patients.

Fasting venous blood samples were collected preoperatively, which were standard practices in our hospital. Within 1–2 h, the blood samples were sent to the Medical Laboratory Center for routine examination. The levels of WBC, CRP, ESR, albumin, globulin, AGR, plasma D-dimer, plasma fibrinogen, PC, MLR, NLR, and PLR were analyzed. Antibiotic use in patients was delayed by at most 2 weeks until after intraoperative specimens were collected, unless the patients needed anti-infective therapy urgently, in which case they were excluded. At least two (usually five) tissue specimens were cultured intraoperatively for at least 7 days after collection and even for 14 days in case of suspected infection associated with low-virulence or specific pathogens.

### Statistical analysis

Categorical variables were expressed as frequencies and percentages and analyzed by Pearson’s chi-square test or Fisher’s exact test. Kolmogorov–Smirnov (KS) test was used to identify whether the data variables were normally or non-normally distributed. Continuous normally distributed variables were presented as mean ± SD (standard deviation), and analyzed by Student’s *t*-tests. Non-normally distributed data were presented as median (IQR), and Mann–Whitney U test was used to analyze numerical variables with non-normal distribution or unequal variance. All differences were considered significant at a value of *p* < 0.05. Receiver operating characteristic (ROC) curves were plotted to determine the diagnostic value of each biomarker for assessing infected nonunion, and the area under the curve (AUC) and 95% confidence interval (CI) were calculated to compare different biomarkers. Based on the AUC value, the discriminatory capacity was interpreted as excellent (0.9–1), good (0.8–0.89), fair (0.7–0.79), or poor (0.6–0.69), and values of 0.5–0.59 indicated that the marker had no discriminatory capacity. The Youden’s index (*J* = [sensitivity + specificity] − 1) was used to determine the optimal predictive cut-offs for calculating AUC. The sensitivity, specificity, positive predictive value (PPV), and negative predictive value (NPV) of each test was calculated. All statistical analyses were performed using STATA version 18.0.

## Results

Basic demographics and clinical characteristics of the included patients among two groups are shown in Table 1. There was no significant difference in age, BMI, smoking history, and site of nonunion between the two groups (*p* > 0.05). In comparison with aseptic cases, there were more male patients (*p* = 0.032) and a higher number of previous open fractures in the infected nonunion group (*p* < 0.001).

**Table 1:** Patient characteristics for the two groups

	Infected group (*n*, 74)	Aseptic group (*n*, 201)	*p* value
Number of men	61/13 (82.4%)	142/59 (70.6%)	0.032*
Age (year, mean ± SD)	47.2 ± 14.8	44.9 ± 14.1	0.249
BMI (kg/m^2^, mean ± SD)	21.2 ± 1.7	23.4 ± 1.3	0.191
Smoking history	28/46 (37.8%)	59/142 (29.4%)	0.212
Previous open fracture	32/42 (43.2%)	27/174 (13.4%)	< 0.001*
*Site of nonunion*			
Clavicle	1/73 (1.4%)	13/188 (6.5%)	0.122
Humerus	9/65 (12.3%)	34/167 (16.9%)	0.454
Radius and/or ulna	2/72 (2.7%)	10/191 (5.0%)	0.542
Femur	21/53 (28.4%)	49/152 (24.4%)	0.543
Tibia	40/34 (54.1%)	91/110 (45.3%)	0.221
Fibula	1/73 (1.4%)	4/197 (2.0%)	1.000

*BMI* body mass index

^*^p < 0.05 indicates statistical significance

**Fig. 1:**
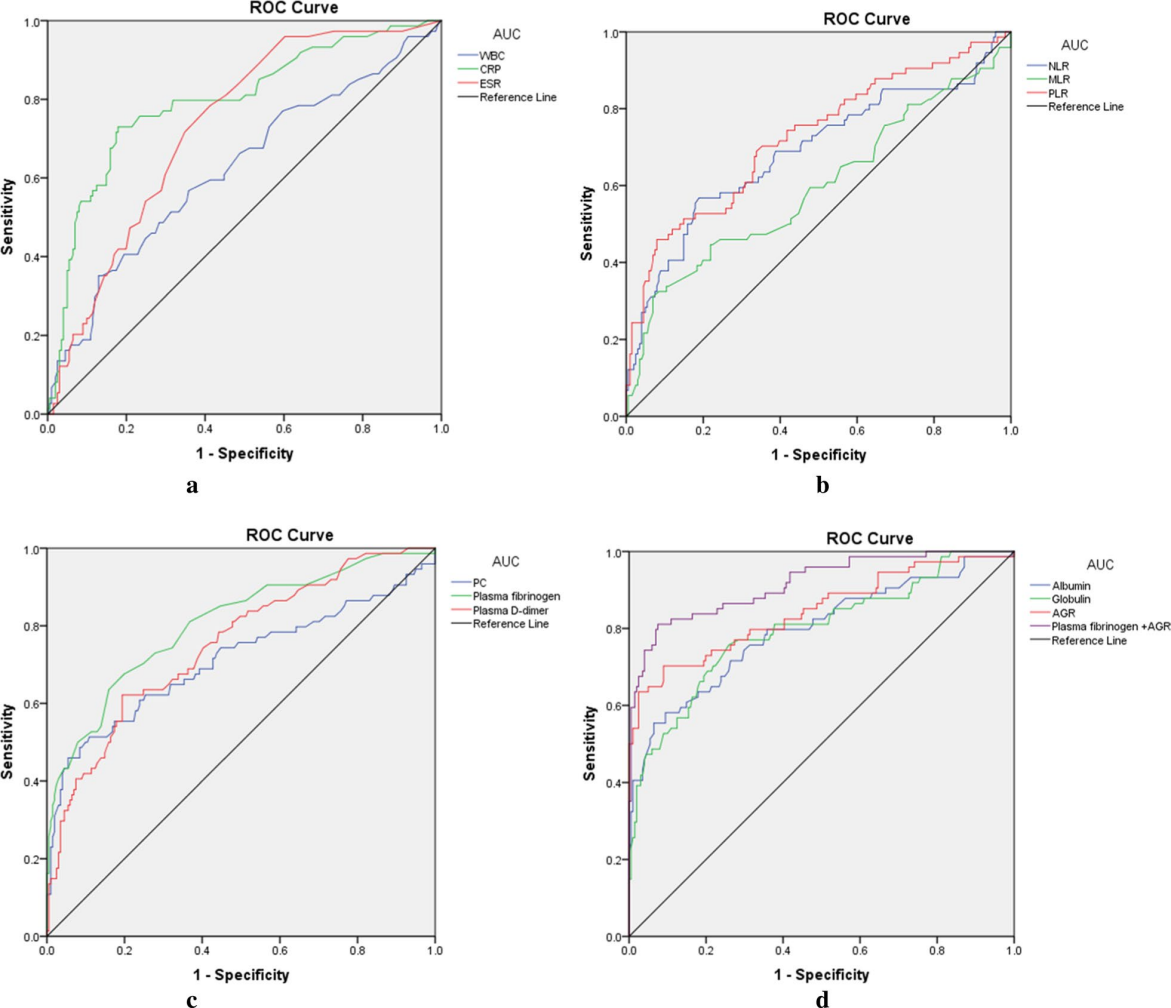
The ROC curves of biomarkers in the diagnosis of infected nonunion. *WBC* white blood cell, *CRP* C-reactive protein, *ESR* erythrocyte sedimentation rate, *PC* platelet count, *MLR* monocyte-to-lymphocyte ratio, *NLR* neutrophil-to-lymphocyte ratio, *PLR* platelet-to-lymphocyte ratio, *AUC* areas under the curve

The WBC, CRP, ESR, NLR, MLR, PLR, PC, plasma D-dimer, plasma fibrinogen, and globulin levels were significantly increased in the infected nonunion group compared with the aseptic group (*p* < 0.05; Table 2). Nonetheless, the albumin and AGR levels of patients in the infected nonunion group were significantly lower than those in the aseptic group (*p* < 0.05). The composite biomarker combining AGR with plasma fibrinogen (0.916) had the highest AUC (Fig. 1 and Table 3). The combination of AGR with plasma fibrinogen showed the highest sensitivity (86.49%), PPV (81.01%), and NPV (94.90%), and the second highest specificity (92.54%).

**Table 2:** Comparison of the tested markers in the two groups

	Infected group (*n*, 74)	Aseptic group (*n*, 201)	*p* value
WBC (10^9^/μL)			0.002*
Median	6.9	6.1	
Interquartile range	5.8–8.4	5.2–7.4	
CRP (mg/L)			< 0.001*
Median	14.1	3.6	
Interquartile range	5.4–29.7	2.5–5.4	
ESR (mm/h)			< 0.001*
Median	17.0	9.0	
Interquartile range	12.0–28.5	5.0–16.5	
NLR			0.016*
Median	2.9	2.0	
Interquartile range	2.0–4.1	1.6–2.5	
MLR			< 0.001*
Median	0.3	0.2	
Interquartile range	0.2–0.4	0.2–0.3	
PLR			< 0.001*
Median	179.3	114.4	
Interquartile range	123.0–233.4	90.3–156.7	
PC (10^9^/L)			< 0.001*
Median	296.0	209.0	
Interquartile range	201.3–384.3	181.0–261.5	
Plasma D-dimer (mg/L)			< 0.001*
Median	1.1	0.4	
Interquartile range	0.5–3.1	0.2–0.8	
Plasma fibrinogen (mg/L)			< 0.001*
Median	3.8	2.7	
Interquartile range	2.9–5.2	2.3–3.2	
Albumin (g/L)			< 0.001*
Median	38.5	41.8	
Interquartile range	35.1–40.5	40.5 = 43.3	
Globulin (g/L)			< 0.001*
Median	32.7	26.9	
Interquartile range	28.4–37.6	23.9–29.6	
AGR			< 0.001*
Median	1.1	1.6	
Interquartile range	1.0–1.5	1.4–1.8	

*WBC* white blood cell, *CRP* C-reactive protein, *ESR* erythrocyte sedimentation rate, *PC* platelet count, *MLR* monocyte-to-lymphocyte ratio, *NLR* neutrophil-to-lymphocyte ratio, *PLR* platelet-to-lymphocyte ratio, *AGR* albumin-to-globulin ratio

All *p* values calculated using Mann–Whitney U test

^*^p < 0.05 indicates statistical significance

**Table 3:** The diagnostic value of tested markers in patients with infected nonunion

Variables	AUC (95% CI)	Optimal cutoff value	Sensitivity (%)	Specificity (%)	PPV (%)	NPV (%)
WBC	0.624 (0.546–0.702)	8.05 × 10^9^/L	37.84	87.06	51.85	79.19
CRP	0.795 (0.732–0.858)	6.5 mg/L	71.62	82.09	59.55	88.71
ESR	0.731 (0.669–0.793)	11.5 mm/h	78.37	58.71	41.13	88.06
NLR	0.687 (0.609–0.765)	2.74	56.76	81.09	52.50	61.22
MLR	0.595 (0.512–0.677)	0.37	31.08	93.03	78.26	67.44
PLR	0.725 (0.653–0.797)	135.12	68.92	66.17	42.86	85.26
PC	0.702 (0.620–0.784)	294.5 × 10^9^/L	51.35	89.05	63.33	83.26
Plasma D-dimer	0.752 (0.686–0.817)	0.92 mg/L	62.16	80.60	78.26	67.44
Plasma fibrinogen	0.805 (0.743–0.867)	3.25 mg/L	67.57	84.08	60.98	87.56
Albumin	0.797 (0.733–0.862)	40.55 g/L	71.62	74.13	50.48	87.65
Globulin	0.795 (0.729–0.862)	32.35 g/L	55.41	93.53	75.93	85.07
AGR	0.845 (0.785–0.904)	1.33	70.27	91.04	74.29	89.27
AGR + Plasma fibrinogen	0.916 (0.876–0.957)	0.89	86.49	92.54	81.01	94.90

*WBC* white blood cell, *CRP* C-reactive protein, *ESR* erythrocyte sedimentation rate, *PC* platelet count, MLR monocyte-to-lymphocyte ratio, *NLR* neutrophil-to-lymphocyte ratio, *PLR* platelet-to-lymphocyte ratio, *AGR* albumin-to-globulin ratio, *AUC* areas under the curve, *CI* confidence interval, *PPV* positive predictive value, *NPV* negative predictive value

## Discussion

Infected nonunion is a major problem in orthopedic and trauma surgery and continues to be a significant clinical challenge [33]. We initially aimed to discover novel infected nonunion-specific biomarkers that can help preoperatively distinguish infected nonunion from other patients with aseptic nonunion after ORIF. A comprehensive review of the literature shows that our study is the first to compare albumin, globulin, AGR, plasma D-dimer, plasma fibrinogen, PC, MLR, NLR, and PLR with traditional inflammatory biomarkers, i.e., WBC, CRP, and ESR, for their ability to screen infected nonunion in patients undergoing reoperation after ORIF. Our research found that preoperative plasma fibrinogen and AGR were reliable blood biomarkers for screening infected nonunion, and the combination of AGR with plasma fibrinogen could further provide more accurate and more specific evaluation for diagnosing infected nonunion in these patients.

Albumin and globulin, easily accessible and reliable biomarkers in the basic metabolic panel, have proven to be critical markers associated with inflammation and infection [34, 35]. Albumin, a negative acute-phase reactant, is widely considered to be an inflammatory and nutritional biomarker in humans [36]. Hypoalbuminemia is correlated with malnutrition, hepatopathy, kidney disease, and all types of inflammation [26, 37]. Globulin, another major serum protein component, consists of various immunoglobulins and acute-phase proteins [38, 39]. Collectively, both decreased albumin and increased globulin played essential roles in response to inflammation and infection functions to dramatically decrease the AGR, suggesting that AGR indicates the body’s inflammatory state more accurately [40, 41]. Previous studies have validated the potential role of globulin and AGR in association with PJI and may serve as potential adjuvant biomarkers in the diagnosis of PJI [29, 30]. In our study, we observed that albumin and AGR were significantly lower, while globulin was considerably higher in patients with infected nonunion than aseptic patients. However, ROC curve analysis revealed that only AGR showed acceptable predictive value for the diagnosis of infected nonunion, with high AUC (0.845), fair sensitivity and PPV (70.27% and 74.29%, respectively), but high specificity and NPV (91.04% and 89.27%, respectively).

Many studies have indicated that systemic and local infections result in fibrinolytic activity, and coagulation-related parameters such as fibrinogen, D-dimer, and PC have been shown to be promising diagnostic biomarkers for the diagnosis of PJI in some studies [17, 42–47]. We found that the AUC of plasma fibrinogen (0.805) was larger than that of plasma D-dimer (0.752) and PC (0.702). However, plasma fibrinogen had a moderate sensitivity and PPV (67.57% and 60.98%, respectively), but a high specificity and NPV (84.08% and 87.56%, respectively).

MLR, NLR, and PLR have been demonstrated as stable and cost-effective biomarkers that reflect the inflammatory response as they mediate inflammation by various biochemical mechanisms [48, 49]. Previous research suggested that NLR may perform better than CRP for diagnosing early PJI [50]. In the contrast, most studies showed that MLR, NLR, and PLR showed limited diagnostic value in the preoperative diagnosis of infected nonunion, which was similar to our result [11, 22]. Our result showed that the diagnostic performance of MLR, NLR, and PLR was limited, as the AUC, sensitivity, and specificity of MLR were 0.595, 31.08, and 78.26%, respectively; those of NLR were 0.687, 56.76, and 52.50%, respectively; and those of PLR were 0.725, 68.92, and 42.86%, respectively.

WBC, CRP, and ESR are the most commonly used biomarkers of infected nonunion. Unfortunately, they are usually affected by other factors such as physiological stress, treatment, and other diseases [7, 51]. Peripheral WBC, CRP, and ESR are typically normal in low-grade infections and afford little value in diagnosing infected nonunion. Moreover, different fractures may have different risks of infection, so it may make sense to analyze these laboratory parameters in the different subgroups of fractures.

This study has some limitations. First, all patients with suspected infected nonunion were collectively analyzed, and thus, these results may not be applicable to all possible subgroups. Second, this study was retrospective, with inherent biases, because electronic medical records may contain incorrect or non-existent information for individual patients. Third, the sample size in our study was relatively small, so we did not include data on any probable effect of antibiotic use or different comorbidities. Last, there are heterogeneities among the patients with different fracture sites. Therefore, multicenter, prospective, comparative studies with larger samples are required to more thoroughly determine the accuracy of these biomarkers for predicting infected nonunion of different fracture sites.

In conclusion, our results show that AGR and plasma fibrinogen may be reliable biomarkers to screen for infected nonunion. The combination of preoperative AGR with plasma fibrinogen may provide more accurate and specific evaluation of infected nonunion. Larger and prospective studies should be carried to verify our findings.

## Data Availability

The final dataset will be available from the corresponding author upon reasonable request.
